# Thiophosphoryl-PMMH Dendrimers for Potential Detection and Remediation of CBRN Contamination: Selected Studies and General Guidelines and Procedures

**DOI:** 10.3390/ma18163805

**Published:** 2025-08-13

**Authors:** Sebastian Lalik, Agnieszka Gonciarz, Robert Pich, Krzysztof A. Bogdanowicz, Witalis Pellowski, Jacek Miedziak, Marcin Szczepaniak, Monika Marzec, Agnieszka Iwan

**Affiliations:** 1 Institute of Physics, Jagiellonian University, Łojasiewicza 11, 30-348 Krakow, Poland; sebastian.lalik@uj.edu.pl (S.L.); monika.marzec@uj.edu.pl (M.M.); 2 Faculty of Security and Safety Research, General Tadeusz Kosciuszko Military University of Land Forces, Czajkowskiego 109, 51-147 Wrocław, Poland; agnieszka.gonciarz@awl.edu.pl (A.G.); robert.pich@awl.edu.pl (R.P.); witalis.pellowski@awl.edu.pl (W.P.); jacek.miedziak@awl.edu.pl (J.M.); marcin.szczepaniak@awl.edu.pl (M.S.); 3 Military Institute of Engineer Technology, Obornicka 136, 50-961 Wrocław, Poland; bogdanowicz@witi.wroc.pl

**Keywords:** phosphorus dendrimers, CBRN, dielectric spectroscopy, thermal imaging, safety engineering

## Abstract

The main idea of this work is to implement organic nanomaterials, such as thiophosphoryl-PMMH dendrimers, for the potential detection and remediation of chemical, biological, radiological, and nuclear (CBRN) contamination. An IR–thermal technique for determining the material specific surface morphology and defects of a thiophosphoryl-PMMH dendrimers is presented. Optical (UV-Vis), thermal (DSC), and electrical (dielectric spectroscopy and thermal imaging) characterizations show that the generation and number of surface groups influence the properties of the investigated dendrimers. Finally, general guidelines and procedures of thiophosphoryl-PMMH dendrimers with various generations are proposed for both civilian and military users.

## 1. Introduction

In the field of safety engineering, innovative materials and technologies supporting environmental protection have been developed in accordance with the principles of Green Chemistry and ecological and energy safety. Nanomaterials offer revolutionary possibilities but require responsible risk management. The key is (i) investing in toxicological studies, (ii) implementing the principles of green nanotechnology (designing safe materials), and (iii) creating international safety standards. Only a balanced approach will maximize benefits while minimizing threats. Nanomaterials, thanks to their unique physical, chemical, and mechanical properties, are used in many fields, bringing significant benefits such as those briefly presented below:

⮚ Medicine and pharmacy: (i) targeted drug delivery: nanoparticles (e.g., liposomes, dendrimers) transport drug substances directly to diseased cells, minimizing side effects; (ii) diagnostics: gold or iron oxide nanoparticles serve as contrast in imaging (MRI, tomography); (iii) cancer therapy: light-activated nanomaterials (photothermia) destroy cancer cells.
⮚ Environmental protection: (i) removal of pollution: titanium dioxide nanofilters break down toxins in water and air when exposed to light; (ii) renewable energy: nanomaterials in solar cells (e.g., perovskites) increase energy conversion efficiency.
⮚ Electronics and industry: (i) miniaturization: graphene, carbon nanotubes or quantum dots enable faster and smaller electronics; (ii) self-cleaning surfaces: TiO_2_ nanoparticle coatings break down organic contaminants when exposed to UV.
⮚ Security and safety engineering: (i) threat detection: nano-sensors identify trace amounts of explosive, chemical or biological substances; (ii) lighter armor: nanocomposites (e.g., nanofibers) strengthen protective materials without increasing weight.

Despite their great potential, nanomaterials carry risks that require caution, such as:

⮚ Health toxicity: (i) effects on cells: some nanoparticles (e.g., carbon nanotubes) can damage DNA or cause inflammation (analogous to asbestos); (ii) bioaccumulation: nanoproducts can accumulate in organisms, disrupting ecosystem functions.
⮚ Environmental hazards: (i) difficulty in recycling: nanomaterials are often not subject to standard disposal processes, leading to contamination; (ii) unpredictable interactions: nanopowders can react with other compounds in the environment, creating toxic mixtures.
⮚ Social and ethical risks: (i) nanotechnological weapons: nanomaterials can be used to create unconventional means of warfare (e.g., toxic aerosols); (ii) technological inequalities: developing countries often lack access to the benefits of nanotechnology, deepening global inequalities.
⮚ Lack of regulation: (i) unknown long-term effects: many nanomaterials have not been tested for health effects after years of exposure; (ii) inadequate standards: current regulations often do not take into account the specifics of nanoparticles, making it difficult to control their use.

Among various types of nanomaterials, such as carbon nanotubes, fullerenes, graphene, aerogels, zeolites, nanoparticles, nanofibers, quantum dots or self-assembling monolayers (SAMs), dendrimers play an important role [1]. Phosphorus-based dendrimers (thiophosphoryl-PMMH) were developed by a group led by Caminade and Majoral [2,3,4,5,6,7,8,9,10,11,12,13,14,15,16,17,18,19,20,21] and are the subject of this work. Considering the functionality, generation, and properties of thiophosphoryl-PMMH, they enable the development of new functional materials with properties such as high dipole moment [3], biocompatibility [10], and good thermal stability [9].

Our interest lies in the properties of thiophosphoryl-PMMH dendrimers for chemical, biological, radiological, and nuclear (CBRN) detection, which is the primary goal of our work. To our knowledge, this issue has not been studied in the literature so far in the context of thiophosphoryl-PMMH dendrimers and their dielectric and thermo-optical properties for CBRN detection. Table 1 shows the key aspects of the beneficial and unfavorable use of nanomaterials, with a particular emphasis on thiophosphoryl-PMMH dendrimers in the context of CBRN protection.

Moreover, we have also analyzed new, extremely toxic chemical substances regarding key threats to public safety. The final outcome of the work is a developed catalogue of threats related to the release of chemical substances in the urban agglomeration. We are currently working on a specialist set for the exploration of forensic traces from places contaminated with CBRN agents. Special protective clothing for work in places contaminated with CBRN agents has been developed, a mobile decontamination chamber has been designed, and preventive protection and first-aid packages have been developed to be used in areas at risk of CBRN agents. This work also aims to determine and estimate the probability and consequences of undesirable events occurring during the performance of official tasks in places contaminated with CBRN. An additional goal was to create procedures for the safe handling of contaminated forensic traces. Despite the potential of known decontamination methods, the problem of the possibility of “destroying” key features of revealed forensic traces during decontamination has still not been solved. Currently, experts are also unable to work with contaminated traces. Therefore, the possibilities of using innovative materials, including nanomaterials, in both the detection and decontamination of damage-sensitive forensic traces, e.g., biological traces, are being considered. The experience gained during the work shows that detection or decontamination techniques using nanomaterials are an alternative to classic solutions, which are not useful due to the potentially destructive nature of the revealed forensic traces [22,23].

The main goal of this work is to implement organic nanomaterials such as thiophosphoryl-PMMH dendrimers for potential detection and remediation of chemical, biological, radiological, and nuclear (CBRN) contamination. Additionally, we propose general guidelines and procedures together with dielectric and IR–thermal studies of dendrimers of various generations. The solutions proposed in this work are dual-use for both civilian and military users.

**Figure 1:**
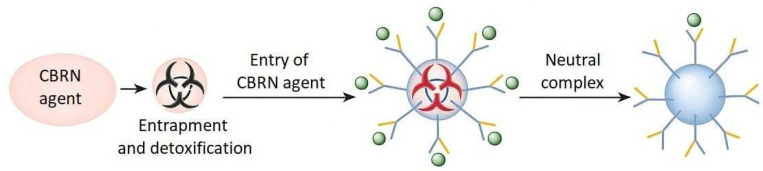
Graphical representation of the idea of our work.

We put forward the hypothesis that higher generation dendrimers, functionalized with specific chemical groups (e.g., thiol, amino, or carboxyl groups), demonstrate higher selectivity, sorption efficiency, and detoxification capabilities for CBRN agents than traditional nanomaterials such as metal oxides or carbon nanotubes, due to their spherical, branched structure and high density of functional surface groups (see Figure 1).

It is well known that dendrimers are a unique class of nanomaterials with a well-defined structure, high symmetry, and multifunctional ends, enabling and exhibiting the following:

- Precise targeting of detection and sorption thanks to surface functionalization (e.g., with compounds that recognize phosphate groups in neurotoxins such as sarin);
- Effective multivalent binding, which increases the strength of interaction with the toxin;
- Chemical selectivity towards various classes of CBRN (e.g., molecules containing metal ions, organophosphate groups, or radioactive isotopes);
- Potential biodegradability, which minimizes the risk of secondary environmental contamination.

Table 2 presents a comparative analysis of selected nanomaterials in terms of their suitability for CBRN. Dendrimers functionalized with specialized ligands offer unique properties that make them the materials of choice for the detection and neutralization of CBRN threats. Their advantage over traditional nanomaterials stems from the synergy between structure, chemically controlled functionalization, and adaptability in biosensor and decontamination systems.

## 2. Materials and Methods

All dendrimers were purchased from Sigma-Aldrich (St. Louis, MO, USA) and were used as received:

- Thiophosphoryl-PMMH-3 dendrimer, generation 0.5 (three aldehyde surface groups); Molecular weight 426; melting point (m. p.) 115–119 °C; purity: 98%; No. CAS: 159213-45-3.
- Thiophosphoryl-PMMH-6 dendrimer, generation 2.0 (six dichlorophosphinothioyl surface groups); Molecular weight 2389.28; m. p. no data; purity: 99%; No. CAS: 173612-59-4.
- Thiophosphoryl-PMMH-6 dendrimer, generation 1.5 (six aldehyde surface groups); Molecular weight 1423.32; m. p. 75 °C; purity: 99%; No. CAS: 169132-80-3.
- Thiophosphoryl-PMMH-12 dendrimer, generation 2.5 (twelve aldehyde surface groups); Molecular weight 3417.21; m. p. no data; purity: 96%; No. CAS: 173612-60-7.

The frequency domain dielectric spectroscopy (FDDS) method was used to study dielectric properties versus frequency of four dendrimer generations. Dielectric spectra versus temperature was measured during cooling down to −20 °C using a broadband impedance Spectrometer Concept 81 (Novocontrol Technologies GmbH & Co. KG, Montabaur, Germany) with temperature accuracy better than 0.5 K. A measuring voltage of 0.25 V was applied, and the measuring frequency range was 10 mHz–10 MHz. The materials studied were placed between two brass electrodes of 9.6 mm diameter; the thickness of samples were measured by micrometer screw and ranged from 0.5 mm to 1.0 mm.

A PerkinElmer DSC8000 differential scanning calorimeter (DSC, PerkinElmer, Waltham, MA, USA) was used to find the degradation temperature of the studied dendrimers. Aluminum crucibles of 30 μL capacity were filled with samples and tightly closed using a press. DSC curves were registered during heating at a rate equal to 10 K/min in the temperature range from −20 °C up to degradation of dendrimers with temperature accuracy better than 0.1 K.

The spectral characteristics in the UV-Vis spectral range (280–800 nm) were recorded on a UV-Vis spectrophotometer Agilent Cary 300 (Agilent Technologies, Santa Clara, CA, USA) with a slit of 0.2 nm and medium scan speed. A capped quartz cuvette was used for all experiments. As received solvent chloroform was used. UV-Vis spectra measurements were performed for the following concentrations of the studied dendrimers: generation 0.5–1.5 × 10^−7^ M, generation 1.5–7 × 10^−7^ M, and generation 2.5–3.5 × 10^−7^ M. For generation 2.0, the UV–Vis spectra measurements were performed as a saturated solution due to the very poor solubility of this compound.

Fourier transform infrared (FTIR) spectra of dendrimers (ca. 1–2 mg) were recorded on an IR Invenio S spectrometer (Bruker, Billerica, MA, USA) with a resolution of 4 cm^−^^1^ and an attenuated total reflectance attachment (ATR) by averaging 64 scans at room temperature (about 20 °C), collected to record the spectra in the range of 4000–400 cm^−^^1^.

A coupled technique composed of a thermographic camera (VIGO cam v50, VIGO System S.A, Ozarów Mazowiecki, Poland) and a multichannel potentiostat–galvanostat (PGStat Autolab M101, Metrohm, Barendrecht, Nederland) was implemented as described in previous work [24]. During the experiment, current values and thermal images were recorded at 3 min intervals and with 0.5 V steps in the range of 0–10 V, as the applied potential increased. The samples were developed from a 20 mg/mL chloroform solution using a spin-coating technique at 900 rpm on an ITO-coated glass support. The results were normalized to rectangular samples of 1 cm^2^. The architecture of a sample was as presented: ITO-coated glass/PMMH layer/silver paste/ITO-coated glass.

## 3. Results

### 3.1. Basic Characteristic of Dendrimers

**Figure 2:**
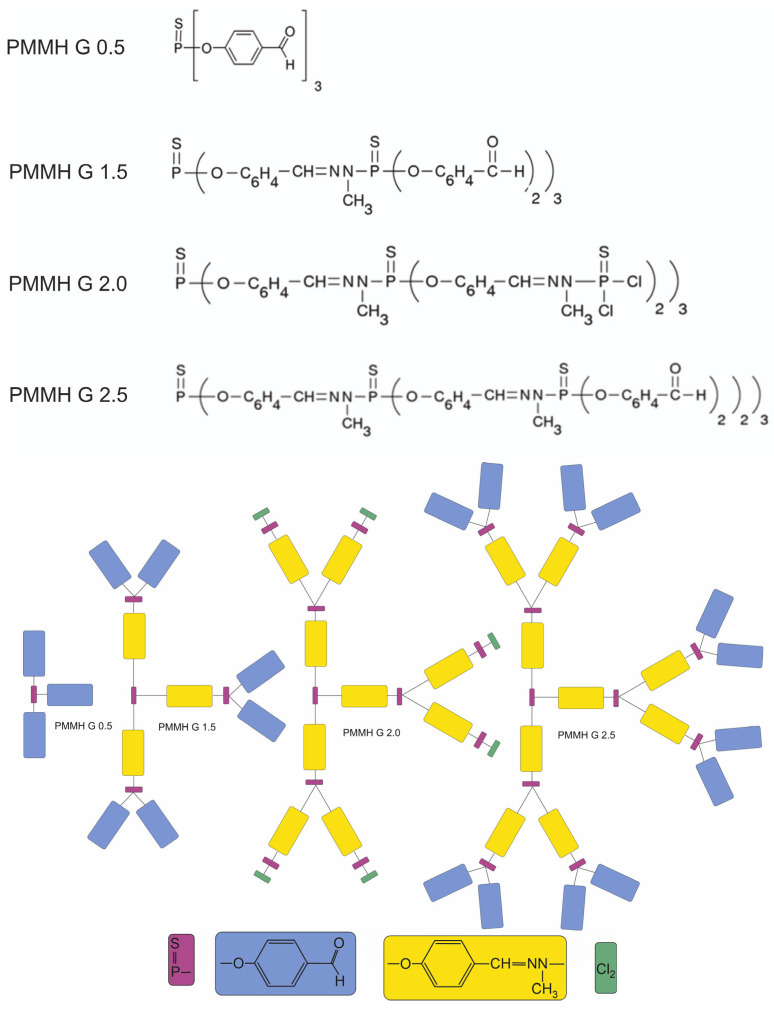
Chemical structure of investigated dendrimers.

Commercially available three thiophosphoryl-phenoxymethyl(methylhydrazono) dendritic cores containing 3, 6, and 12 aldehyde surface groups (Figure 2) and one thiophosphoryl-PMMH-6 dendrimer with six dichlorophosphinothioyl surface groups were first investigated by ATR-FTIR and UV-Vis spectroscopy.

**Figure 3:**
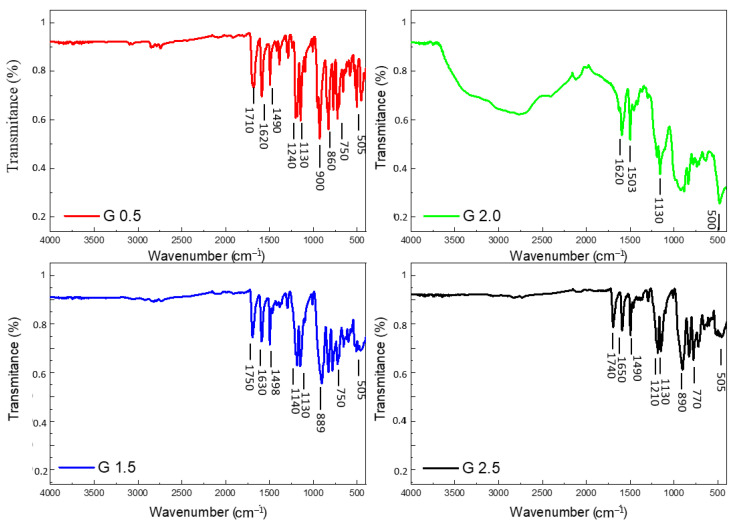
ATR-FTIR spectra of studied dendrimers.

Figure 3 shows the ATR-FTIR spectra registered for the studied dendrimers. The intense band in the IR spectra at ca. 1730 cm^−1^ is evidently assigned to the stretching vibrations of the C=O bond. The spectra band at 1620 cm^−1^ is assigned to the stretching vibrations of the C–C bond of the benzene ring. The band at ca. 1500 cm^−1^ may be assigned to the C=N vibrations mixed with the stretching vibrations of the C–C bonds in the benzene ring. The weak bands at 1460 and 1470 cm^−1^ in the spectra refer to the antisymmetric and symmetric bending vibrations of the CH_3_ group. The strong band at 1240 cm^−1^ is mainly caused by the C–O band stretch. The intensity in the experimental spectra band at 1130 cm^−1^ is assigned to CCH angle bending. The very intense band in the IR spectra at 900 cm^−1^ is caused by the stretch of the N–N bonds. The band at 760 cm^−1^ includes the contributions of the stretching vibrations of the C–O, P=S and P–O bonds. The line at 505 cm^−1^ is assigned to the stretching vibrations of the P=S bond [25].

Comparing the chemical structure of investigated dendrimers shown in Figure 2, it is obvious that the dendrimers with generations 0.5, 1.5, and 2.5 have a similar structure (containing 3, 6, and 12 aldehyde surface groups, respectively), and therefore their IR spectra should also be similar. However, the structure of generation 2.0 differs from the previous ones due to the presence of six dichlorophosphinothioyl surface groups. Therefore, the IR spectrum of dendrimer generation 2.0 differs from the spectra of the other dendrimers studied. Due to the overlap of the dendrimer generations 0.5, 2.0, and 2.5 peaks, we presented the IR spectra of each dendrimer separately in the paper (see Figure 3).

**Figure 4:**
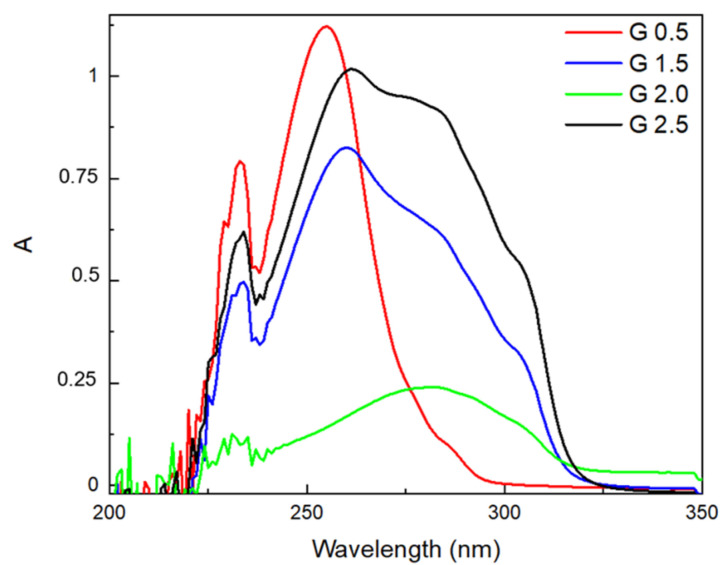
UV-Vis spectra of studied dendrimers.

UV-Vis spectra for studied dendrimers are presented in Figure 4. In chloroform solution, the UV-Vis spectra display one or two more or less well-defined absorption maxima or humps, depending on the generation of the dendrimer. The UV-Vis spectrum of dendrimer generation 0.5 is characterized by two absorption bands at ca. 233 and 255 nm, with a slight hump observed at 287 nm. The UV-Vis spectra of dendrimer generations 1.5 and 2.5 are very similar, with two well-defined maxima at 234 and 260 nm and two humps at ca. 287 and 305 nm. In the UV-Vis spectrum of dendrimer generation 2.0, only one absorption band at 282 nm is well visible. Due to the poor solubility of this dendrimer, its spectrum was performed for a saturated solution.

**Figure 5:**
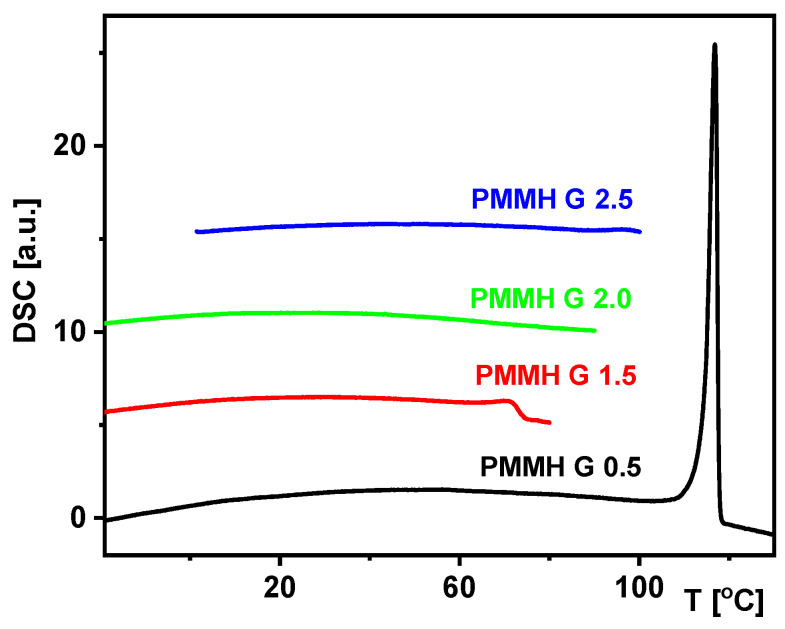
DSC curves registered for studied dendrimers during heating at rate of 10 K/min. Different heating ranges are associated with the different degradation temperatures of the samples studied.

DSC curves registered for the studied dendrimers are presented in Figure 5. It is seen that no anomaly is visible only for generation 2.0, while a small peak was recorded for generations 1.5 and 2.5, and a strong one for generation 0.5. These registered anomalies are connected with the degradation of dendrimers. The results of DSC measurements were used to plan the FDDS measurements.

### 3.2. Dielectric Spectroscopy

**Figure 6:**
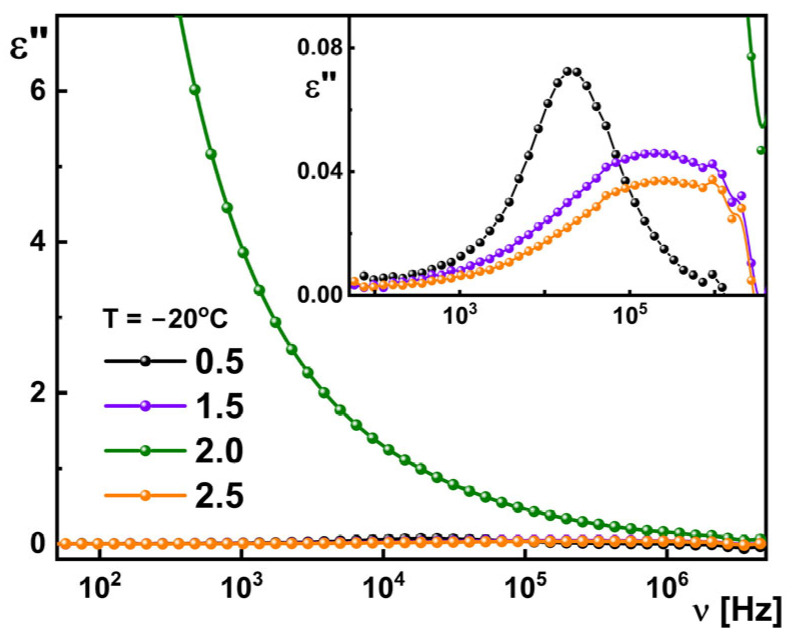
Dielectric absorption for the studied dendrimers registered at −20 °C. The solid lines are just lines to guide the eye.

Dielectric absorption of the studied dendrimers registered for temperature −20 °C are presented as an example in Figure 6. It is seen that even for generations 0.5, 1.5, and 2.5, one anomaly is visible; they differ significantly in both shape and relaxation frequency. Meanwhile, for generation 0.5 one narrow, symmetric process is visible with relaxation frequency equal to 18 kHz; for generations 1.5 and 2.5, the visible relaxation process is broad and shifted towards higher frequencies (relaxation frequency is ca. 200 kHz). The dielectric spectra for generations 1.5 and 2.5 are very similar. No relaxation process was registered for generation 2.0.

**Figure 7:**
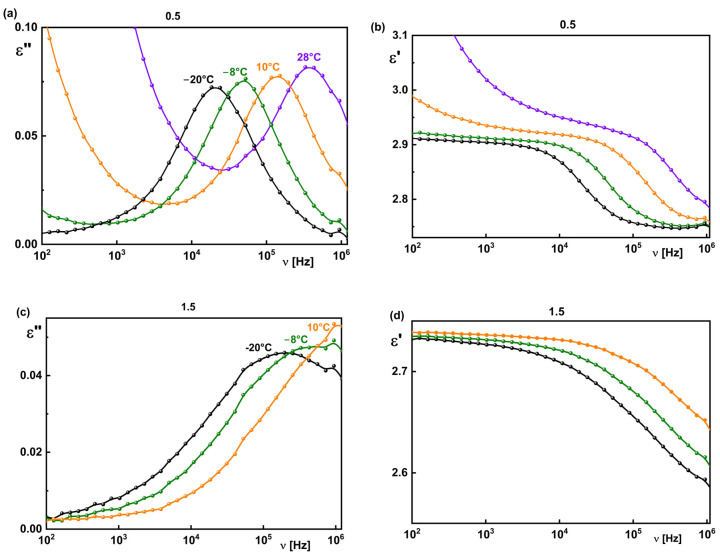
Dielectric absorption (**a**,**c**) and dispersion (**b**,**d**) registered at chosen temperatures for PMMH G 0.5 and G 1.5. Legend in (**a**) is valid for (**b**) while legend in (**c**) is valid for (**d**).

Evolution of the dielectric spectra with temperature for generations 0.5 and 1.5 is presented as an example in Figure 7. Although the process registered for generation 0.5 has a different character than that for generation 1.5 and exists in the broader temperature range, they behave with temperature in the same way: the relaxation frequency of the visible relaxation process decreases with decreasing temperature for both generations 0.5 and 1.5, while its intensity almost does not change.

The same evolution with temperature of the dielectric spectra as for generation 1.5 was found for generation 2.5. The Cole–Cole model was fitted to the dielectric spectra [26]:

$$\varepsilon *=\;\varepsilon_{\infty }+\;\frac{\Delta \varepsilon }{{1+\;\left(\;i\omega \tau_{r}\right)}^{1-\alpha }}-i\frac{\sigma (\omega )}{\varepsilon_{0}\omega }$$

where *ω* is measuring frequency, $∆\varepsilon =\varepsilon_{0}-\varepsilon_{\infty }$ is dielectric increment, $\varepsilon_{\infty }$ is dielectric dispersion at high frequency limit, $\varepsilon_{0}$ is dielectric dispersion at low frequency limit, *α* is the distribution parameter of relaxation time, τr=12πνr is relaxation time, $\nu_{r}$ is relaxation frequency, and *σ*(*ω*) is conductivity.

**Figure 8:**
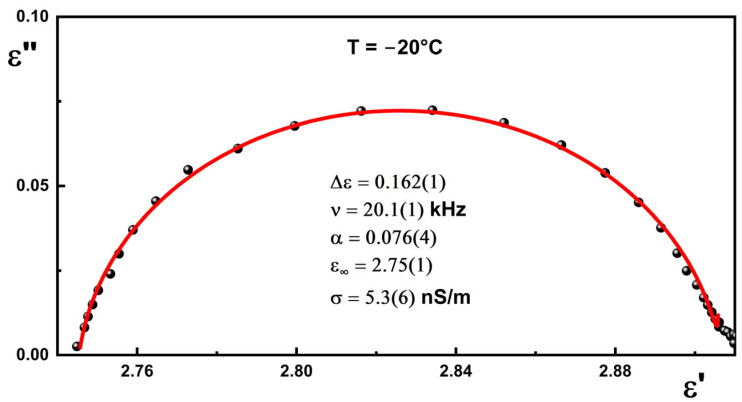
Cole–Cole diagram for generation 0.5 registered at −20 °C. Solid lines are the result of fitting Cole–Cole formulae to the experimental data.

As an example, the dielectric spectrum as a the Cole–Cole diagram with fitting result for generation 0.5 is presented in Figure 8.

### 3.3. Thermo-Electric Studies

The purpose of the coupled technique measurements was to assess the use of PMMH dendrimer as an organic conductive layer in a detection system for the possible detection of CBRN agents. During the experiment, the current response and thermal images were recorded by applying an external potential to a sample with a sandwich architecture, where the studied layer was placed between two ITO-coated glass substrates, one of which was coated with conductive silver paste. In this study, four different generations of PMMH dendrimers, representing various structures and molecular masses, were investigated. Our previous work has proven that this technique can provide information regarding the reorientation of molecules and help in the identification of degradation processes occurring in situ during the exposure to temperature during the self-healing process [27].

**Figure 9:**
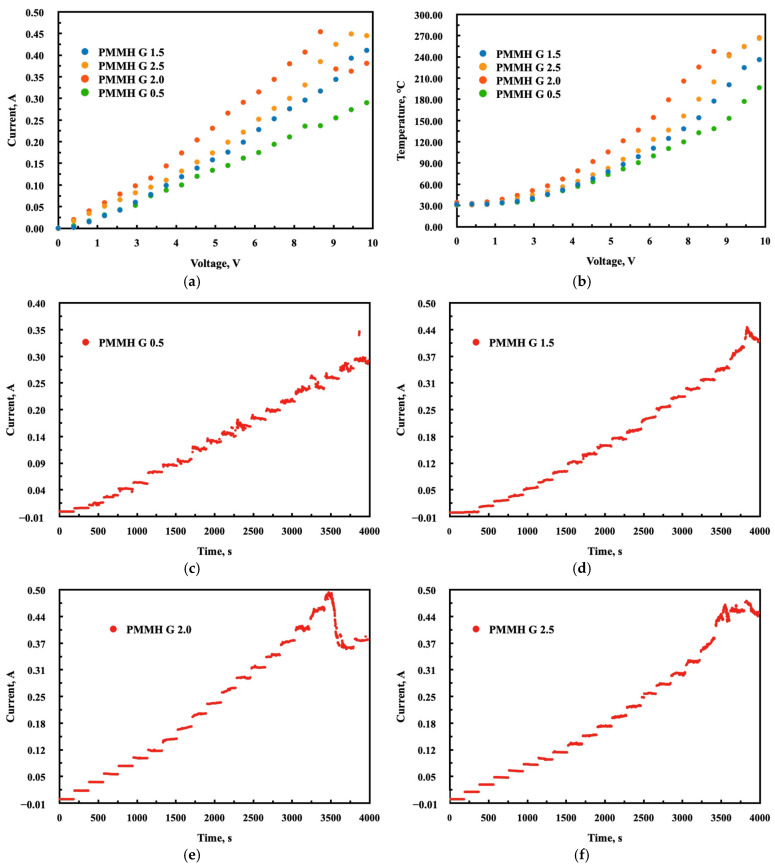
Relation between current (**a**) and temperature (**b**) and applied potential and current change over time for PMMH generations 0.5, 1.5, 2.0, and 2.5 (**c**–**f**), respectively.

For all the samples, sheet resistance values were obtained within the range of approximately 22 Ω/cm^2^ to 35 Ω/cm^2^, in the order of G 2.0 > G 2.5 > G 1.5 > G 0.5. All samples displayed a stable increase in current flow over time, with minor fluctuations, up to 9.0 V (see Figure 9). The PMMH G 0.5 showed the most significant fluctuation, which may suggest a reorientation of the molecules within the layer, given its smallest molecular size. For potentials above 9.0 V, the current reading loses stability due to the physical disintegration of the sample setup. No degradation signals were observed throughout the whole experiment. All the samples demonstrated very high resistance to temperature, reaching values above 220 °C, except for G 0.5, for which the highest observed temperature was slightly below 200 °C.

**Figure 10:**
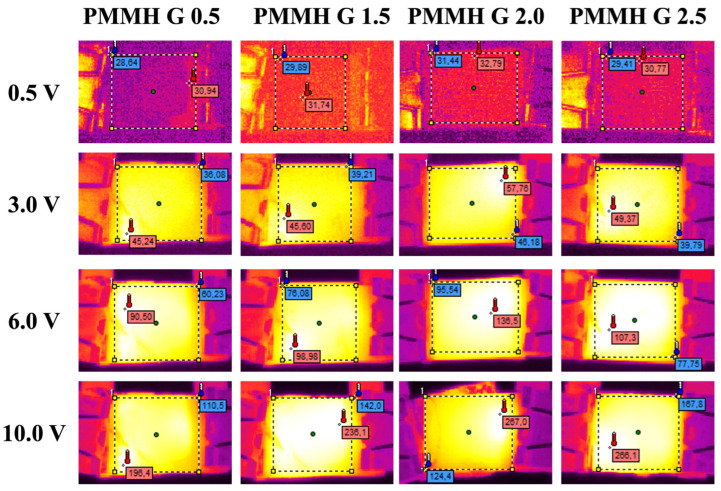
Thermographs registered at 0.5 V, 3.0 V, 6.0 V, and 10.0 V for PMMH generations 0.5, 1.5, 2.0 and 2.5, respectively.

The analysis of thermal images registered during the incremental application of potential revealed that, in all cases, the heat distribution was uniform without any discernible structural defects (Figure 10).

## 4. Discussion

**Figure 11:**
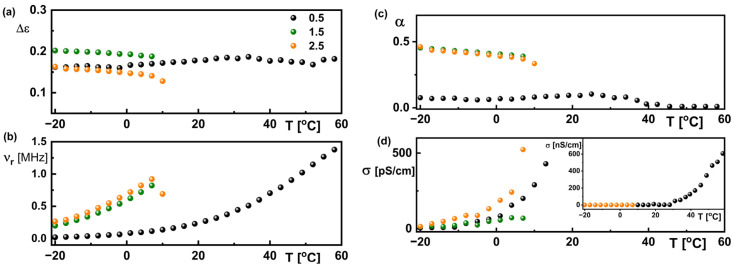
Temperature dependence of the dielectric increment (**a**), the relaxation frequency (**b**), the distribution parameter of relaxation time (**c**), and the specific electric conductivity (**d**) for all sample studied obtained as a result of fitting the Cole–Cole formulae to the experimental data. Legend in (**a**) is valid for all graphs.

As a fitting result, the temperature dependence of relaxation frequency and dielectric increment was found for all samples studied (Figure 11a,b). It is seen that the relaxation process was revealed in the wide temperature range only for generation 0.5 (from −20 °C up to +61 °C), while for another two generations (1.5 and 2.5) it was in the range between −20 °C and +10 °C, although the degradation of these materials is above +60 °C. This process goes beyond the measuring frequency range with increasing temperature. The relaxation frequency for the generation 0.5 sample strongly changes with temperature above 10 °C.

The recorded dielectric process is certainly related to the monomer forming generation 0.5, denoted in Figure 2 as a blue rectangle. This monomer is not modified in generations 1.5 and 2.5. In generation 2.0, this monomer does not exist (it is modified) and the process is not visible. Since there are three free monomers in generation 0.5, a strong process is visible in a wide temperature range. This process is much stronger and occurs at lower frequencies than that recorded for generations 1.5 and 2.5, because the molecules of these generations are much more complex (Figure 2). This complexity of molecules broadens this process (the distribution parameter of relaxation time is much larger for generations 1.5 and 2.5 than for 0.5; see Figure 11c) and shifts it towards higher frequencies in the temperature range from −20 °C to +10 °C. As was mentioned above, the relaxation frequency of this process increases above 10 °C for generation 0.5, reaching values comparable or even higher than those for generations 1.5 and 2.5 (Figure 11b). On the other hand, its dielectric increment is almost the same in all studied samples; it is approximately 0.2 and changes only slightly with temperature (Figure 11a). The specific electric conductivity of all samples studied does not differ significantly below 0 °C and is several dozen pS/cm. In turn, it increases rapidly above 10 °C for generation 0.5 and reaches about 600 nS/cm (Figure 11d).

**Figure 12:**
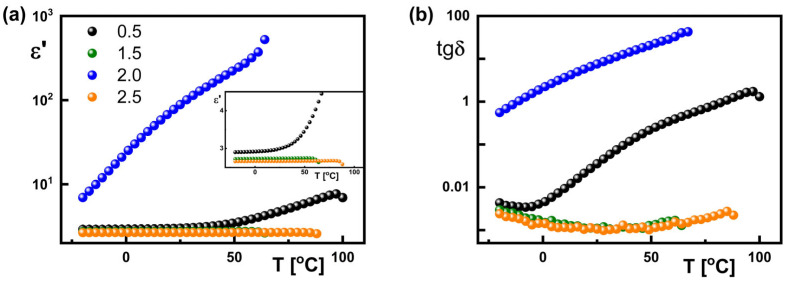
Temperature dependence of the dielectric constant (**a**) and dielectric loss (**b**) at 1 kHz registered for all for generations studied. Legend in (**a**) is valid for (**b**).

When considering the applications of dielectric materials, an important parameter is both the value of the dielectric constant (according to definition—dielectric dispersion at 0 kHz) and the dielectric loss (loss of energy, e.g., heat, which is described by the tangent of the loss angle). Figure 12 presents the temperature dependence of the dielectric constant ε′ and dielectric loss tgδ at 1 kHz, registered for all generations studied. It is visible that the dielectric constant ε′ (1 kHz) decreases with decreasing temperature for all studied dendrimers; however, the mentioned decrease is practically imperceptible for generations 1.5 and 2.5, while it is the greatest for generation 2.0. Moreover, dielectric constant ε′ (1 kHz) initially increases and then there is a monotonic decrease with decreasing temperature for generations 0.5, 1.5, and 2.5, which is not observed for generation 2.0.

In turn, tgδ changes strongly with temperature for generations 0.5 and 2.0, while for generations 1.5 and 2.5 it is practically the same and changes only slightly with temperature (amounting to about 0.01 in the entire temperature range). On the other hand, the dielectric constant ε′ (1 kHz) at a given temperature for the studied dendrimers decreases with the increasing complexity of their chemical structure. As an example, the dielectric constant and the dielectric loss for all studied dendrimers at a temperature of 22 °C are gathered in Table 3. It is visible that for generation 2.0 the dielectric constant ε′ is the highest, about 25 times higher than for generation 0.5. It is similar for dielectric loss, which is three times higher for generation 2.0 than for generations 1.5 and 2.5.

Such a small dielectric constant ε′ (1 kHz) and dielectric loss tgδ (1 kHz) at 22 °C for generations 0.5, 1.5, and 2.5 (Figure 12, Table 3) are comparable to the values for linear dielectric polymers such as polycarbonate, poly(phenylene sulfide), poly(ethylene 2,6-naphthalate), and polystyrene [28]. Therefore, it seems that the dendrimers of generations 0.5, 1.5, and 2.5 are an alternative for linear dielectric polymers from the dielectric point of view. In addition, low dielectric loss tgδ is characteristic for other dielectric linear polymers, such as PET (poly(ethylene terephthalate)) or POFNB (polyoxafluoronorbornene), while high tgδ (as for generation 2.0) is similar to that for the dielectric nonlinear polymer P(VDF-CTFE), but for this polymer ε′ is about six times smaller than for generation 2.0. As it turns out, the studied dendrimers are characterized by a similar dielectric constant ε′ and low dielectric loss tgδ as organic polymers.

### Proposal of General Guidelines and Procedures for the Use of Dendrimers for the Potential Detection and Remediation of CBRN Contamination

The unique dielectric and thermoelectric properties of thiophosphoryl dendrimers play a key role in their electrochemical and catalytic applications, particularly in the context of detecting and neutralizing CBRN agents. The mechanism of action and the advantages of these dendrimers in these tasks are presented in Table 4.

Very important for the practical use of PMMH dendrimers in the context of CBRN detection and remediation are the general guidelines and procedures, as presented in Table S1 in Supporting Information [29,30,31]. The thermo-electric studies also confirmed the possibility of their implementation in sensors, where the PMMH compounds can be used as a conductive matrix deposited directly on an electrode. Depending on the desired agent for the detection, the chemical structure of the dendrimer can be modified chemically, thanks to the presence of chlorine and carboxyl groups [32]. The literature [33,34,35] reports focus on the strategy of detoxication or sequestration reactions that lead to the incorporation of the Novichok agent into the dendrimer and the release of halogens as a method to reduce neurotoxicity. The same mechanism will occur during contact with PMMH dendrimers; however, the expected outcome of the interaction between the sensing layer and the agent will be the alteration of the electric properties registered by impedance and DC conductivity. Our point of view of PMMH dendrimers in CBRN applications is presented in Table 5 [36,37,38,39,40,41,42,43,44,45,46,47,48,49,50,51,52,53].

Taking into consideration various aspects of this work, a comparative report evaluating PMMH dendrimers compared to leading CBRN detection and remediation platforms, focusing on performance metrics like limit of detection (LOD), response time, and operational stability, are presented in Table 6. The evaluation identifies areas of superiority and weakness relative to key alternatives such as MOF composites, metal-oxide nanofibers, and enzyme-based gels.

Additionally, it should be stressed that PMMH dendrimers are perfect for the following:

- Sensitivity: Fluorescently modified PMMH dendrimers achieve sub-ppb LODs, outperforming most MOF or metal-oxide platforms.
- Speed: Sub-minute fluorescence response allows real-time monitoring in the field.
- Chemical Stability: They maintain integrity over wide temperature and pH ranges, crucial for military and CBRN deployment.
- Design Versatility: PMMH dendrimers are highly modifiable (via phosphonates, thiols, carboxyls), allowing custom affinity for various CBRN agents.
- Integration Compatibility: Can be deposited as thin films, nanogels, or embedded into textiles—superior platform flexibility.

Moreover, PMMH dendrimers currently have limitations such as the following:

- Catalytic Degradation Efficiency: While functionalized dendrimers can neutralize agents, they do not match enzymatic detox efficiency (e.g., OPH hydrolysis of organophosphates).
- Bioactivity Risks: Despite promising in vitro biocompatibility, long-term bioaccumulation or degradation byproducts remain under study.
- Scalability: PMMH dendrimers synthesis at the dendrimer level is more complex and cost-intensive than mass-produced MOF powders or nanofibers.

PMMH dendrimers offer a compelling hybrid of sensing specificity, chemical stability, and integration ease, unmatched by many traditional nanomaterials. However, for rapid biocatalytic neutralization, enzyme-based systems still set the benchmark. Future hybrid systems (e.g., PMMH with OPH enzyme conjugates) may combine the strengths of both platforms.

## 5. Conclusions

Thiophosphoryl-functionalized PMMH dendrimers present a very promising platform for the detection and remediation of chemical, biological, radiological, and nuclear (CBRN) threats. Their unique dendritic architecture provides a high surface area and abundant reactive sites that enable precise molecular recognition and efficient functionalization with fluorescent, catalytic, or selective binding moieties. These properties enable the development of highly sensitive, selective, and tunable nanosensors for CBRN agents, in particular organophosphorus compounds such as sarin or VX.

Studies have demonstrated the capacity of such dendrimers to serve as both passive detectors and active scavengers, capable of binding and neutralizing toxic compounds. Their compatibility with various substrates and delivery systems (e.g., coatings, sprays, hydrogels, and nanocomposites) further enhances their applicability in field-deployable CBRN defense systems.

Despite their clear potential, further research is needed to evaluate their long-term stability, biocompatibility, and environmental safety. Integration into operational CBRN response protocols also requires standardized testing and alignment with international safety guidelines.

In conclusion, thiophosphoryl-PMMH dendrimers represent a cutting-edge nanotechnological solution for modern CBRN defense. Through continued multidisciplinary development and validation, they may significantly improve early threat detection, rapid response capability, and overall public safety.

Based on dielectric studies, it turns out that the dendrimers of generations 0.5, 1.5, and 2.5 behave similarly, while generation 2.0 behave in a different way. The relaxation process observed for generations 0.5, 1.5, and 2.5 is certainly related to the monomer forming generation 0.5 and existing in both generations 1.5 and 2.5. Moreover, the studied dendrimers are characterized by a similar dielectric constant ε′ and low dielectric loss tgδ to linear dielectric polymers. Therefore, it seems that the dendrimers of generations 0.5, 1.5, and 2.5 are an alternative for linear dielectric polymers from a dielectric point of view.

## Figures and Tables

**Table 1:** Key aspects of the beneficial and unfavorable use of nanomaterials with particular emphasis on thiophosphoryl-PMMH dendrimers in the context of CBRN.

Category	Guidelines/Procedures	Benefits	Risks/Limitations
Synthesis and preparation	Controlled polymerization (e.g., ATRP) for the construction of the PMMH core.	Highly repeatable structure.	High cost of synthesis.
Contamination detection	Functionalization with thiophosphoryl groups (reaction with PSCl_3_ in anhydrous medium).	Possibility of modification for specific threats.	Risk of contamination by toxic precursors (e.g., chlorides).
Contamination removal	Functionalization with fluorescent tags (e.g., rhodamine) for optical detection of toxins.	High sensitivity (ppb level detection).	Sensitivity to environmental interferences (e.g., pH, temperature).
Safety	Integration with electrochemical sensors.	Fast response (less than 1 min).	Limited adsorption capacity at high concentrations of contaminants.
Deactivation and disposal	Use of dendrimers as adsorbents in filters or gels.	Simultaneous neutralization of chemicals and pathogens.	Potential bioaccumulation of non-degraded dendrimers in ecosystems.

**Table 2:** A comparative analysis of selected nanomaterials in terms of their suitability for CBRN.

Feature	Dendrimers (np. PAMAM)	Carbon Nanotubes	Metal Oxides (e.g., TiO_2_, ZnO)
Functionalizable	High, precise	Difficult, limited	Variable
Density of functional groups	Very high	Low–Medium	Average
Selectivity of detection	High (ligand-specific)	Low	Low-Moderate
Sorption capacity	High (3D structure)	Moderate	Moderate
Ability to neutralize toxins	High (chemical reactivity)	Limited	Variable
Biodegradability/Toxicity	Potentially biodegradable	Biopersistentne	Often toxic or insoluble
Can be used in bio-sensors	Very good	Limited	Good (but less selective)

**Table 3:** Dielectric constant and dielectric loss at 1 kHz for all studied dendrimers at of 22 °C.

PMMH G	0.5	1.5	2.0	2.5
ε′ (1 KHZ)	2.98	2.74	77.42	2.66
TANδ (1 KHZ)	0.028	0.001	6.612	0.001

**Table 4:** The mechanism of action and the advantages of PMMH dendrimers in electrochemical CBRN identification and catalytic degradation of CBRN agents.

Tasks	Dielectric Properties: Electrochemical CBRN Identification	Thermoelectric Properties: Catalytic Degradation of CBRN Agents
Mechanism	Thiophosphoryl dendrimers contain electronegative phosphate and sulfur groups (P=S, P–SH), which influence the local polarity and dielectric constant of the material. High local permittivity favors the concentration of the electric field around target molecules (e.g., organophosphorus neurotoxins such as sarin), facilitating their electrochemical detection.	Under the influence of local heating (e.g., solar radiation or microwave pulse), PMMH dendrimers with thiophosphoryl groups generate temperature gradients, which can initiate charge transfer (Seebeck effect). This facilitates the formation of reactive oxygen species or sulfide anions, which catalytically degrade CBRN toxins.
Benefits	Increased detector sensitivity—changes in impedance or surface potential are more pronounced in the presence of CBRN molecules. Selectivity—P–S and P–O bonds can specifically interact with ester and phosphate groups in toxins. Signal stability—the branched structure of the dendrimer enables stable and reproducible signaling in electrochemical sensors (e.g., EIS, CV).	Photocatalytic degradation of, for example, halogen derivatives or mustard agents (HD), Self-heating sensor layers—detection + neutralization in a single material, Electron and ion mobility—supports the breakdown of ester, organophosphate, or thiol bonds in toxins.

**Table 5:** PMMH dendrimers in CBRN applications.

Material Characteristics		
Action	Scientific Rationale	Ref.
Structural Analysis (NMR, FTIR, MS)	It allows confirmation of the presence of phosphorus and thiophosphoric groups responsible for chemical activity	[36]
Chemical and thermal stability assessment	PMMH withstands extreme temperatures and pH—crucial in battlefield conditions	[37]
Toxicology tests (in vitro/in vivo)	A prerequisite for operational safety and compliance with REACH regulations	[38]
Functional modification		
Addition of detection groups (e.g., fluorophores, VX ligands)	Increases selectivity and the ability to quickly detect CWA	[39]
Surface modification	It enables better affinity for toxic molecules, e.g., due to electrostatic charges	[40]
Smart sensor design	Fluorescence signaling after CBRN contact increases immediate detection	[41]
Contamination detection		
Application on active surfaces (electrodes, gels)	Dendrimers can be permanently deposited on substrates for field applications	[42]
Sensor sensitivity calibration	Detection below 10 ppb—essential for trace amounts of CWA	[43]
Integration with mobile systems (UAVs, robots)	Remote threat detection enhances operator safety	[44]
Decontamination procedures		
Coating of carrier materials (e.g., fabrics, foams)	In situ, mustard gas neutralization confirmed in <10 min	[45]
Application in the form of gels or liquids	Easy application to equipment, possibility of decontamination without specialized tools	[46]
Disposal of waste materials	ISO and EPA compliant—no secondary toxin emissions	[47]
Evaluation of efficacy and safety		
Performance Validation	Proven efficacy in simulated and operational settings	[48]
Monitoring of side effects	Observation of human/environmental impacts required by regulations	[49]
Operator Training	Reducing the risk of errors and increasing effectiveness on the ground	[50]
Integration with CBRN systems		
Compliance with guidelines (e.g., NATO AEP-66)	It guarantees interoperability with existing military and civilian systems	[51]
Institutional cooperation (OPCW, WHO, CDC)	Ensures the implementation of best practices and international compliance	[52]
Early warning systems (AI and sensors)	Ability to integrate with IoT and threat prediction systems	[53]

**Table 6:** Comparative performance report: PMMH dendrimers vs. current CBRN platforms. (own study based on literature research).

Metric	PMMH Dendrimers	MOF Composites (ZIF-8, UiO-66-NH_2_)	Metal-Oxide Nanofibers (e.g., ZnO, TiO_2_)	Enzyme-Based Gels (e.g., OPH, PTE Hydrogel Systems)
LOD (VX, Sarin, Chlorine)	<1 ppb (fluorogenic probes)	1–10 ppb (depends on linker type)	5–50 ppb (gas phase)	1–5 ppb (liquid phase)
Response Time	<30 s (fluorescent response)	1–5 min	~3 min	<1 min (if active enzyme present)
Operational Stability	High (−20 to 80 °C, pH 2–11)	Moderate (humidity sensitive)	High (excellent heat resistance)	Poor (loss of activity >40 °C)
Shelf Life	>12 months (dry state)	~6–9 months (desiccant needed)	~12 months	~1–3 months (requires refrigeration)
Signal Specificity (CB vs. non-CB)	High (via functional ligands)	Medium–High	Low (non-specific oxidation)	High (active site-selective)
Ease of Integration (sensors)	High (thin films, nanogels)	Medium (requires scaffolding)	High (electrospun mats)	Low (gel matrix not field robust)
Detoxification Capability	Moderate–High (thiol groups)	Moderate (passive adsorption)	Low–Moderate (oxidation only)	High (enzymatic hydrolysis)
Reusability	Yes (up to 10 cycles)	Limited (~3–5 cycles)	Yes (cleaning between uses)	No (single-use or biohazardous)

## Data Availability

The original contributions presented in this study are included in the article and Supplementary Materials. Further inquiries can be directed to the corresponding author.

## Supplementary Materials

The following supporting information can be downloaded at: https://www.mdpi.com/article/10.3390/ma18163805/s1, Table S1: Proposed general guidelines and procedures for the use of PMMH dendrimers in the context of CBRN detection and remediation.
