# Supplementary material for: Thiophosphoryl-PMMH Dendrimers for Potential Detection and Remediation of CBRN Contamination: Selected Studies and General Guidelines and Procedures
Source: Materials (Basel). 2025 Aug 13;18(16):3805. doi: 10.3390/ma18163805 (PMC12387595; doi:10.3390/ma18163805)
Supplement: Supplementary file 1 [file materials-18-03805-s001.zip › materials-3736392-supplementary.pdf]

Supporting Information

Thiophosphoryl-PMMH dendrimers for potential detection and remediation of CBRN contamination: selected studies and general guidelines and procedures

Sebastian Lalik[1], Agnieszka Gonciarz[2], Robert Pich[2], Krzysztof A. Bogdanowicz[3], Witalis Pellowski[2], Jacek Miedziak[2], Marcin Szczepaniak[2], Monika Marzec[1], Agnieszka Iwan[2*]

1 Institute of Physics, Jagiellonian University, Łojasiewicza 11, 30-348 Krakow, Poland.
2 Faculty of Security and Safety Research, General Tadeusz Kosciuszko Military University of Land Forces, Czajkowskiego 109, 51-147 Wrocław, Poland.
3 Military Institute of Engineer Technology, Obornicka 136, 50-961 Wrocław, Poland.

Correspondence: agnieszka.iwan@awl.edu.pl

Table S1. Proposed general guidelines and procedures for the use of PMMH dendrimers in the context of CBRN detection and remediation

**Material characterization and preparation**	**Functional and structural analysis:** Before using PMMH dendrimers, their structure and functionality should be analyzed in detail, using techniques such as NMR spectroscopy, FTIR, and mass analysis.	**Stability assessment:** Checking the chemical and thermal stability of dendrimers under operational conditions is crucial to ensure their effectiveness in real CBRN scenarios.	**Biocompatibility and toxicity testing:** Conducting in vitro and in vivo tests to assess the potential biological responses and toxicity of PMMH dendrimers.
**Functionalization and modification of dendrimers**	**Introduction of detection groups:** Integration of fluorescent tags or ligands specific for CBRN agents (e.g. sarin, VX) to increase sensor selectivity and sensitivity.	**Surface modification:** Tailoring the surface of dendrimers to improve their interaction with specific CBRN threats.	**Creating "smart" sensors:** Designing dendrimers capable of changing the signal (e.g. fluorescence) in the presence of specific CBRN agents.
**Contamination detection procedures**	**Application to measuring surfaces:** Applying PMMH dendrimers to electrodes, gels, or other materials to create active sensing surfaces.	**Sensor calibration:** Determining detection thresholds and sensor sensitivity under controlled conditions.	**Implementation in mobile systems:** Integration of dendrimer sensors with drones, robots or portable field monitoring devices.
**Decontamination procedures**	**Coating of carrier materials:** Creating active surfaces by coating materials with PMMH dendrimers, enabling neutralization of CBRN agents.	**Application in the form of nanogels or liquids:** Use of dendrimers in the form of gels or liquids to decontaminate surfaces and equipment.	**Waste collection and disposal:** Safe disposal of materials containing neutralized CBRN agents in accordance with applicable regulations.
**Evaluation of effectiveness and safety**	**Method validation:** Regular testing of detection and decontamination efficiency using PMMH dendrimers.	**Monitoring for adverse reactions:** Observation of potential side effects of dendrimers use, both on humans and the environment.	**Staff training:** Preparing operators to safely and effectively use PMMH dendrimers in CBRN emergency situations.

**Integration with CBRN systems**	**Guideline Compliance:** Alignment of procedures with current guidelines and standards for responding to CBRN threats.	**Cooperation with institutions:** Coordination of activities with appropriate agencies and institutions responsible for security and response to CBRN threats.	**Development of early warning systems:** Integration of dendrimer sensors with CBRN threat monitoring and warning systems.
